# Experience of flexible bronchoscopy in the pediatric pulmonary diseases clinic

**DOI:** 10.5578/tt.202401827

**Published:** 2024-03-26

**Authors:** Mehmet Mustafa ÖZASLAN, Gökçen KARTAL ÖZTÜRK, Meral BARLIK, Ece OCAK, Fevziye ÇOKSUER, Bahar GİRGİN DİNDAR, Atacan ÖĞÜTÇÜ, Ece HALİS, Esen DEMİR, Figen GÜLEN

**Affiliations:** 1 Department of Pediatric Pulmonary Diseases, Ege University Faculty of Medicine, İzmir, Türkiye

## Abstract

**ABSTRACT**

**
Experience of flexible bronchoscopy in the pediatric pulmonary diseases clinic
**

**Introduction:** *Flexible bronchoscopy is a valuable method in the diagnosis and treatment of respiratory tract diseases in children. This study aimed to examine the indications for and results of flexible bronchoscopy in children.
*

**Materials and Methods:** *The study included patients aged 0-18 years who underwent flexible bronchoscopy between 1 January 2017 and 31 December 2022. The patients were evaluated for demographic characteristics, indica- tions for bronchoscopy, comorbidities, bronchoscopy findings, and the results of bronchoalveolar lavage.
*

**Results:** *During the defined study period, a total of 410 flexible bronchoscopy procedures were performed. 51.9% of the patient population were male, and 48.1% were female, with a mean age of 96.93 ± 63.45 months. The most common indication for flexible bronchoscopy was recurrent lower respiratory tract infection (26.8%), followed by chronic cough (19.1%). The bronchoal- veolar lavage culture results showed that the most commonly isolated micro- organisms were H. influenzae non-type b (7.8%) followed by M. catarrhalis (7.3%). Mucus obstruction and secretion (33.0%) constituted the most com- mon bronchoscopic findings, while the flexible bronchoscopy examination was normal in 27% of patients. No serious complications occurred in any patient during or after the procedure.
*

**Conclusion:** *The results of this study demonstrated that the most common indication for flexible bronchoscopy was recurrent lower respiratory tract infection and the most common bronchoscopy finding was purulent secretion with mucus obstruction. Flexible bronchoscopy is an important diagnostic and treatment tool for patients with recurrent respiratory symptoms. It is a highly valuable method as it enables direct visualization of the airways and facilitates the collection of bronchoalveolar lavage samples.
*

**Key words:** *Flexible bronchoscopy; bronchoalveolar lavage
*

**ÖZ**

**
Çocuk göğüs hastalığı kliniğimizde fleksible bronkoskopi deneyimlerimiz
**

**Giriş:** *Fleksible bronkoskopi çocuklarda solunum yolu hastalıklarının tanı ve
*

*
tedavisinde kullanılan değerli bir yöntemdir. Bu çalışmada amacımız çocuklar- da fleksible bronkoskopi endikasyonlarını ve sonuçlarını araştırmaktır.
*

**Materyal ve Metod:** *Çalışmaya çocuk göğüs hastalıkları bilim dalımızda 1 Ocak 2017 ile 31 Aralık 2022 tarihleri arasında 0-18 yaş arasında fleksible bronkoskopi yapılan hastalar dahil edildi. Hastaların demografik özellikleri, bronkoskopi yapılma endikasyonu, eşlik eden komorbiditeler, bronkoskopi bulguları ve bronkoalveolar lavaj sonucu kaydedildi.
*

**Bulgular:** *Toplam 410 FB işlemi uygulanmıştı. Hastaların %51,9’u erkek, ortalama yaşları 96,93 ± 63,45 aydı. Fleksible bronkoskopi- nin en sık endikasyonu tekrarlayan alt solunum yolu enfeksiyonu (%26,8), ikinci en sık endikasyon kronik öksürük (%19,1) idi. Bronkoalveolar lavaj kültür sonucu en sık üreyen mikroorganizma H. influenzae non-tip b (%7,8) iken ikinci sıklıkla M. catarrhalis (%7,3) idi. En sık bronkoskopik tanı mukus tıkacı ve sekresyon (%33,0) iken, hastaların %29,7’sinin fleksible bronkoskopisi normal- di. İşlem esnasında ve sonrasında ciddi bir komplikasyon gelişen hasta olmadı.
*

**Sonuç:** *Çalışmamızda en sık FB endikasyonu tekrarlayan alt solunum yolu enfeksiyonu iken en sık bronkoskopi bulgusu pürülan sekresyon ve mukus tıkacıydı. Fleksible bronkoskopi, tekrarlayan solunum semptomları olan hastalarda önemli bir tanı ve tedavi ara- cıdır. Hava yollarının direk görüntülenebilmesi ve bronkoalveolar lavaj örneğinin alınabilmesi ile çok değerli bir yöntemdir.
*

**Anahtar kelimeler:** *Fleksible bronkoskopi; bronkoalveolar lavaj
*

## INTRODUCTION

Flexible bronchoscopy (FB), which is widely used in the diagnosis and treatment of respiratory tract diseases, provides the opportunity to visualize upper and lower airways, thereby allowing anatomic and dynamic information about the airways to be obtained (1). The main aim of pediatric flexible bronchoscopy continues to be diagnostic (2). The diagnostic indications include stridor, chronic cough, recurrent wheezing, recurrent lower respiratory tract infections, structural anomalies, hemoptysis, and radiological anomalies (2,3). As a result of the evaluation of the upper respiratory tract, functional and structural airway anomalies such as choanal atresia, subglottic stenosis, laryngeal cyst, and vocal cord dysfunction can be diagnosed with FB (3). However, in children, this procedure requires deep sedation (3).

Samples for diagnostic purposes can be obtained by advancing as far as the distal ends of the lungs with the bronchoalveolar lavage procedure. Saline, which is soluble in alveolar fluid is administered to the distal airways and then withdrawn. The cell ratios on the alveolar surface can be measured in the fluid, and cytological and microbiological studies can be performed (4-6). Microbiological tests of cultures, polymerase chain reaction (PCR), and cell count analysis can be made. For diagnostic purposes, hemosiderin-loaded macrophages, lipid-loaded macrophages, and immunological tests can be performed.

For both diagnosis and treatment, bronchoscopy is important in immunocompromised patients (7). Appropriate samples can be obtained from cystic fibrosis patients, which can guide antibiotic treatment (7,8). Whole lung lavage is another method of obtaining samples from the lungs and is used in pulmonary alveolar proteinosis treatment. Although

rigid bronchoscopy is accepted as the gold standard for the removal of foreign bodies, flexible bronchoscopy can also be used (9).

Usually, no complications are seen after the procedure, and the most common airway complications that may be encountered are laryngospasm and rupture. Therefore, patients must be closely monitored and oxygen support should be given.

This study aimed to investigate the indications for flexible bronchoscopy performed in our center and to examine the bronchoscopy results.

## MATERIALS and METHODS

Approval for this retrospective study was granted by the Ethics Committee of the Medical Faculty (decision no: 23-11T/37). Written consent was obtained from all patients and their parents. The study included patients aged 0-18 years who underwent flexible bronchoscopy between January 2017 and December 2022. The demographic characteristics, indications for bronchoscopy, bronchoalveolar lavage fluid culture results, comorbidities, and bronchoscopy findings were recorded for all the patients.

The bronchoscopy procedure was performed using a

2.8 mm diameter bronchoscope for infants and young children (BF-XP160F Evis Exera, Olympus, Japan) and a 3.2 mm diameter bronchoscope for older children (BF-3C Evis Exera, Olympus, Japan). Written informed consent was provided by the parents of all the children before the procedure. The bronchoscopy procedure was performed in the operating theatre after patients fasted for six hours. Airway safety was ensured using either a laryngeal mask or an endotracheal tube, depending on the patient’s clinical status. The bronchoalveolar lavage procedure was performed by instilling 1 mL/kg of

saline three times for patients weighing <20 kg, and with 20 mL of saline for patients weighing >20 kg.

All FB procedures were performed by a senior specialist in pediatric chest diseases. The bronchoalveolar lavage (BAL) samples were taken from the right middle lobe and the left lingular segment (9,10). In patients with localized atelectasis and findings of infection, samples were taken from the lobes where the findings were detected radiologically. The diagnosis of airway malacia was made from visual examination during the bronchoscopy in spontaneous respiration (11,12). The bronchoscopy secretion type was classified as type 1, type 2, or type 3 secretion. Type 1 is defined as no secretion in less than a third of all the bronchi, type 2 as secretions filling between one-third and two-thirds of all the bronchi, and type 3 as secretions filling more than two-thirds of all the bronchi. According to the bronchoscopic secretion score, six grades were defined; grade 1: no secretion, grade 2: bubbles in less than half of the bronchi, grade 3: type 1 secretion in less than half of the bronchi, grade 4: type 1 secretion in more than half of the bronchi or type 2 secretion in less than half of the bronchi, grade 5: type 2 secretion in more than half of the bronchi, grade 6: type 3 secretion in less than half of the bronchi. Throughout the procedure, the heart rate, blood pressure, and oxygen saturation rates of all the patients were continuously monitored.

As this was a retrospective study, patients with incomplete information in the files were excluded from the study.

## Statistical Analysis

Statistical analysis was performed using SPSS software (version 23.0; IBM SPSS Statistics). Values are expressed as mean ± standard deviation for continuous variables, median and range for nonparametric data, and number and percentage (%) for categorical variables.

## RESULTS

During the defined study period, a total of 410 flexible bronchoscopy procedures were performed. The patient population consisted of 213 (51.9%) males and 197 (48.1%) females with a mean age of

96.93 ± 63.45 months. The most common indication for flexible bronchoscopy was recurrent lower respiratory tract infection (n= 110, 26.8%), followed by chronic cough (n= 78, 19.1%), persistent

atelectasis (n= 74, 18.1%), hemoptysis (n= 40,

9.8%), and suspected foreign body (n= 22, 5.4%). FB was performed using a laryngeal mask in 392 (95.6%) cases, an intubation tube in 10 (2.4%), and a tracheostomy cannula in eight (2.0%). Comorbidities were most frequently determined as immune deficiency (6.1%), followed by cystic fibrosis (5.6%), and congenital airway malformation (3.9%). The treatments administered during FB include n-acetylcysteine (n= 74, 18%), dornase alpha (n= 18,

4.4%), and fresh frozen plasma (n= 1, 0.2%). Whole lung lavage was performed on a patient diagnosed with pulmonary alveolar proteinosis.The demographic characteristics of the patients and the indications for FB are presented in Table 1.

The FB procedure was performed on 110 patients with recurrent lower respiratory tract infections. Following bronchoscopy, the most common diagnoses were persistent bacterial bronchitis (n= 32, 29.0%), mucus obstruction and purulent secretion (n= 28, 25.5%), and anatomic defect (n= 15, 13.6%). The most commonly identified microorganism in the bronchoalveolar lavage fluid of this group was *H. influenzae non-type b* (n= 11, 34.4%). The bronchoscopy results were reported as normal in 20% of the patients. The most common findings were mucus obstruction (n= 135, 33.0%), bronchomalacia (n= 72, 17.5%), congenital lower airway anomaly (n= 23, 5.6%), and mucosal inflammation-granulation (n= 14, 3.4%). The most frequently seen secretion type was type 1 (n= 238, 58.0%), followed by type 2 (n= 110, 26.8%). The bronchoscopic findings of secretion types are shown in Table 2. In 62.7% of the patients with mucus obstruction, the radiological imaging completely recovered following bronchoscopy.

FB was performed on 78 patients due to chronic cough, and the results were normal in 41% of these patients. Persistent bacterial bronchitis was determined in 12 (15.4%) patients, anatomic defect in 10 (12.8%), and foreign body aspiration in four. The most commonly identified pathogen in the bronchoalveolar lavage fluid of patients with chronic cough was *M. catarrhalis *.

FB was performed on 25 patients due to immune deficiency. The most common findings on the radiological imaging of these patients were peribronchial thickening, ground-glass appearance, hilar-mediastinal lymphadenopathy, and atelectasis.

**Table:** 

**Table 1.** Demographic characteristics of patients	
**Sex (male, %)**	213 (51.9%)
**Age (mount), median ± SD**	96.93 ± 63.45
** Indications for Flexible Bronchoscopy, n (%) **	
Recurrent lower respiratory tract infection	110 (26.8)
Chronic cough	78 (19.1)
Persistent atelectasis	74 (18.1)
Hemoptysis	40 (9.8)
Suspicion of foreign body aspiration	22 (5.4)
Persistent wheezing	21 (5.2)
Microbiological sample	15 (3.7)
Suspected anatomical disorder	14 (3.5)
Difference ventilation in the lung	9 (2.1)
Suspected interstitial lung disease	9 (2.1)
Etiology of bronchiectasis	8 (1.9)
Persistent stridor	6 (1.4)
Vascular ring airway compression	3 (0.7)
Before tracheostomy closure	1 (0.2)
**Comorbidity, n (%)**	
Immunodeficiency	25 (6.1)
Cystic fibrosis	21 (5.6)
Congenital airway malformation	16 (3.9)
Hematopoietic stem cell transplant	12 (2.9)
Congenital heart disease	9 (2.2)
Malignity	8 (1.9)
Neurological disease	6 (1.4)
Metabolic disease	3 (0.7)
Primary ciliary dyskinesia	3 (0.7)
Tuberculosis	2 (0.5)
Pulmonary alveolar proteinosis	1 (0.2)
** How flexible bronchoscopy is performed, n (%) **	
Laryngeal mask	392 (95.6)
Intubation tube	10 (2.4)
Tracheotomy cannula	8 (2.0)
** Treatment administered during bronchoscopy **	
N-acetylcysteine	74 (18.0)
Dornase alpha	18 (4.4)
Fresh frozen plasma	1 (0.2)

The most commonly identified pathogen in the bronchoalveolar lavage fluid of these patients was

*S. aureus* (62.1%). When the bronchoalveolar lavage results were examined, there was no growth in the cultures of 56.6% of the patients. The three most

commonly isolated microorganisms were

*H. influenzae non-type b* (n= 32, 7.8%), *M. catarrhalis* (n= 30, 7.3%), and *S. pyogenes* (n= 26, 6.3%). The bronchoalveolar lavage culture results are shown in Table 3. In one patient with a recurrent lung infection,

**Table:** 

**Table 2.** Bronchoscopy results		
**n (%)**		
**Flexible Bronchoscopy Findings**		
Normal findings	82	(20.0)
Mucus plug	135	(33.0)
Bronchomalacia	72	(17.5)
Congenital lower airway anomaly	27	(6.6)
Congenital upper airway anomaly	23	(5.6)
Mucosal inflammation/laceration/granulation	14	(3.4)
Tracheomalacia	12	(3.0)
Foreign body aspiration	11	(2.7)
Gastroesophageal reflux	10	(2.4)
Laryngomalacia	9	(2.2)
Airway abnormality in more than one region	7	(1.7)
Subglottic web	4	(1.0)
Other	4	(1.0)
**Secretion Type**	1	(0.2)
Type 1	238	(58.0)
Type 2	90	(22.0)
Type 3	82	(20.0)
**Bronchoscopic Secretion Score**	16	(3.9)
Grade 1	110	(26.8)
Grade 2	86	(21.0)
Grade 3	75	(18.3)
Grade 4	81	(19.7)
Grade 5	35	(8.5)
Grade 6	23	(5.7)

**Table:** 

**Table 3.** Bronchoalveolar lavage culture results	
**Culture Result**	**n (%)**
Culture negative	232 (56.6)
* Haemophilus influenzae non-type b *	32 (7.8)
*Moraxella catarrhalis*	30 (7.3)
*Streptococcus pyogenes*	26 (6.3)
*Streptococcus pneumoniae*	22 (5.4)
*Pseudomonas aeruginosa*	12 (2.9)
*Staphylococcus aureus*	10 (2.4)
*Escherichia coli*	8 (2.0)
*Candida albicans*	6 (1.5)
*Aspergillus fumigatus*	5 (1.2)
*Stenotrophomonas maltophilia*	4 (1.0)
*Mycobacterium bovis*	1 (0.2)
**BAL liquid DNA**	
*Cytomegalovirus*	2 (0.5)

*P. aeruginosa* was isolated in the BAL fluid, and as the sweat test indicated high levels, the patient was referred for genetic analysis and subsequently diagnosed with cystic fibrosis.

The FB procedure was performed on 40 patients due to hemoptysis, and 12.5% of these patients had a diagnosis of cystic fibrosis. Other diagnoses were post-infectious hemoptysis (12.5%), foreign body aspiration (5%), tuberculosis (2.5%), hydatid cyst

(2.5%), and Heiner syndrome (2.5%).

Of the 22 patients who underwent FB due to suspected foreign body aspiration, a foreign body was determined in 11 (50%) patients. These foreign bodies were dried fruit/nuts in seven patients, a piece of a toy in two, part of a pen in one, and paper packaging in one. These were all removed with rigid bronchoscopy by a pediatric surgeon.

No major complications were observed in any patient during or after the procedure. During the procedure, there was a drop in oxygen saturation in

15 (3.6%) patients. In three of these cases, the procedure was terminated, and in 12 the procedure was continued after the oxygen saturation level was restored.

## DISCUSSION

There is increasing use of FB in children for diagnostic and treatment purposes. Although FB is an invasive procedure, the complication rate is low. Bronchoalveolar lavage fluid is highly valuable for diagnosis. It is a versatile test as it allows for culture, PCR, pathology, cytology, and immunological tests to be conducted. The use of FB is recommended in recurrent, prolonged conditions such as recurrent lower respiratory tract infections and chronic cough. This study aimed to present the indications for and results of flexible bronchoscopy performed in our center over six years.

The mean age of the patients in this study was 96.93

± 63.45 months, and the most common indication for FB was recurrent lower respiratory tract infection (26.8%), followed by chronic cough (19.1%). In a previous large-scale study conducted in Türkiye, the mean age of patients was 84 months, and the most common indication for FB was reported to be recurrent pneumonia (17%) (13). Consistent with the current study, a study reported that recurrent respiratory tract infection (32.2%) was the most common indication for bronchoscopy (14). Recurrent

respiratory tract infections are among the most common reasons for presentation at the pediatric thoracic diseases outpatient clinic, with various etiological factors contributing to this phenomenon. FB should be performed for diagnostic purposes in cases with recurrent lower respiratory tract infections and etiology that cannot be determined with existing diagnostic methods.

The second most common indication for bronchoscopy in the current study was chronic cough (19.1%). The study reported that 32% of bronchoscopy indications were tracheal, laryngeal, or chronic wet cough (14). A chronic cough is an important finding of respiratory tract diseases in children. The most frequent causes of chronic cough include asthma, post-infection cough, and bronchiectasis. Patients with a suspected diagnosis and for whom the chronic cough cannot be improved with medical treatment should be evaluated with FB. The European Respiratory Society (ERS) guidelines recommend airway bronchoscopy for patients with a persistent chronic wet cough following four weeks of appropriate antibiotic use (15). If non-invasive sampling cannot be achieved in children with lower respiratory tract infections unresponsive to antibiotic treatment, bronchoscopy is recommended (16,17). The isolation of the causative agent in patients with chronic cough guides the selection of antibiotics and treatment.

The laryngeal mask is the most commonly utilized method for ensuring airway safety and has been proven to be effective and safe. The American Thoracic Society has recommended that airways can be better evaluated during spontaneous respiration and the procedure should be continued afterward with a laryngeal mask (LMA). Additionally, the use of LMA is associated with a lower risk of abdominal distension and aspiration. Bronchoscopy can be performed using an endotracheal intubation tube, but it carries a greater risk of hypoxemia and hypercarbia. Especially in critical patients with poor cardiopulmonary reserves, hypoxemia can develop due to decreased tidal volume, intrapulmonary shunt (V/Q incompatibility), and the frequent aspiration and administration of saline for BAL, which washes away the surface active substance and causes alveolar overflow. In the current study, LMA was used in 95.6% of the patients, and a drop in oxygen saturation was seen in 3%, but no major complication developed in any patient.

The most common bronchoscopy finding in the current study was mucus obstruction with purulent secretion. The variability in its determination across different studies could be attributed to the differences in selected patient groups. The bronchoscopy results of the current study can be attributed to the most common bronchoscopy indications being determined as recurrent pneumonia and chronic cough. Mucus obstruction and purulent secretion, commonly associated with chronic disease, can also be indicative of conditions such as foreign body aspiration or poorly treated pneumonia. These presentations may result in radiological evidence of atelectasis, necessitating FB in such cases.

Of the 22 FB procedures performed in this study due to suspected foreign body aspiration (FBA), a foreign body was identified in 50%, all of which were subsequently removed by a pediatric surgeon using rigid bronchoscopy. If there is a history of FBA, even if the physical examination findings are normal, FB should be performed in suspected patients. FBA can cause various clinical conditions ranging from recurrent lung infections to asphyxia and death. The most common symptoms are obstruction, dyspnea, cough, stridor, and cyanosis. Occasionally, subcutaneous emphysema may develop. In 40% of patients, there is no history of FBA. In the physical examination, there may be a decrease in respiratory sounds in the lung where the foreign body has settled, and rales and rhonchus can be heard in other areas. Rigid bronchoscopy continues to be the gold standard in the treatment of FBA in children (18).

The most common comorbidity in this study was immune deficiency. In patients with atelectasis, persistent consolidation on radiological images, and particularly in those with recurrent pneumonia, identifying the causative agent guides medical treatment decisions. Agents that do not grow in standard cultures may grow in BAL fluid. The most common indication in patients with immune deficiency was determined to be lower respiratory tract infection. In a study by Efrati et al., the most common indication for FB in patients with immune deficiency was reported to be neutropenic fever and respiratory problems (19). Various infectious agents may be implicated in patients with immune deficiency, and effectively clearing secretions can be highly beneficial in the clinical management and follow-up of these individuals.

In 43.4% of the bronchoalveolar lavage fluid cultures, microbial growth was observed. The most commonly identified microorganism was *H. influenzae non-type*

*b.* The growth of microorganisms may be affected by previous antibiotic use, particularly in cases where no growth is observed. A previous study reported that

*S. aureus* and *H. influenzae non-type b* were the most commonly isolated microorganisms in BAL fluid (20). Microorganisms may exhibit variability from center to center, potentially attributed to differences in antibiotic resistance profiles and duration of antibiotic use. The type of microorganism detected in BAL fluid can vary depending on the patient’s comorbidities and the duration of antibiotic use.

When bronchoscopy complications are examined, hypoxemia is the most frequently seen complication. Although FB is a safe procedure, studies in the literature have reported the most common complications to be hypoxia, bleeding, infection, and pneumothorax (21,22). Fever following bronchoscopy is a temporary complication that may occur and is often seen in children aged <2 years (23). In the current study, fever developed after the procedure in two patients, but resolved within 24 hours.

For the flexible bronchoscope to be used on another patient after one procedure, it must undergo thorough manual disinfection according to appropriate standards, followed by high-level disinfection. The personnel performing the procedure must have completed training and undergo annual proficiency tests. Personal protective equipment must be used during the procedure and when handling the bronchoscope afterward.

## Study Limitations

The main limitation of this study was its single-center, retrospective design. Additionally, due to the retrospective nature of the study, patients with incomplete records were excluded from the study.

## CONCLUSION

In conclusion, flexible bronchoscopy is a procedure with few complications that can be safely performed in children. It continues to be increasingly utilized for diagnostic purposes in children. In cases with prolonged respiratory tract problems such as recurrent lower respiratory tract infection, chronic cough, persistent atelectasis, and persistent wheezing, the

evaluation of the airway with flexible bronchoscopy and obtaining a BAL sample can be extremely beneficial in reaching a diagnosis. FB should be performed in patients with persistent radiological findings and respiratory complaints and in those with chronic diseases such as cystic fibrosis or immune deficiency. The results of this study revealed that the most frequent indication for FB was recurrent lower respiratory tract infection, with the most common bronchoscopic findings being purulent secretion and mucus obstruction, followed by airway malacia. There is increasing use of FB in the diagnosis and treatment of respiratory tract diseases in children.

**Ethical Committee Approval:** This study was approved by the Ege University Medical Researches Ethics Committee (Decision no: 23-11T/37, Date: 02.11.2023).

## CONFLICT of INTEREST

The authors declare that they have no conflict of interest.

## AUTHORSHIP CONTRIBUTIONS

Concept/Design: MMÖ, GKÖ, FG Analysis/Interpretation: MMÖ, FÇ

Data acqusition: MMÖ, EO, AÖ, MB, EO Writing: MMÖ, BGD, ED, FG

Clinical Revision: MMÖ, GKÖ, FG, ED Final Approval: MMÖ, FG, ED

## REFERENCES

1. *
  Faro A, Wood RE, Schechter MS, Leong AB, Wittkugel E, Abode K, et al. American Thoracic Society ad hoc com- mittee on flexible airway endoscopy in children. Official American Thoracic Society technical standards: Flexible airway endoscopy in children. Am J Respir Crit Care Med 2015; 191(9): 1066-80. https://doi.org/10.1164/ rccm.201503-0474ST*
2. *
  Tschiedel E, Eckerland M, Felderhoff-Mueser U, Dohna- Schwake C, Stehling F. Sedation for bronchoscopy in children: A prospective randomized double-blinded trial. Pediatr Pulmonol 2021; 56(5): 1221-9. https://doi. org/10.1002/ppul.25235*
3. *
  Bhat JI, Charoo BA, Tramboo ZM, Zahoor S. Proceduralist given sedation is safe and feasible in pediatric flexible bronchoscopy. J Pediatr Intensive Care 2019; 8(4): 214-7. https://doi.org/10.1055/s-0039-1692173*
4. *
  Sinha V, Gurnani D, Barot DA. A study of applications of rigid bronchoscopy in pediatric patients. Indian J Otolaryngol Head Neck Surg 2014; 66(2): 142-4. https:// doi.org/10.1007/s12070-013-0667-1*
5. *
  Wood RE, Daines C. Bronchoscopy and bronchoalveolar lavage in pediatric patients. In: Wilmott RW, Bush A, Boat TF eds. Kending and Chernick’s Disorders of the Respiratory Tract in Children. Eight edition. Philadelphia: Saunders, 2012; 94-109. https://doi.org/10.1016/B978- 0-7216-3695-5.50011-0*
6. *
  Goussard P, Pohunek P, Eber E, Midulla F, Di Mattia G, Merven M, Janson JT. Pediatric bronchoscopy: Recent advances and clinical challenges. Expert Rev Respir Med 2021; 15(4): 453-75. https://doi.org/10.1080/17476348*

*.2021.1882854*

7. *
  Liu H, Sarkar S, Azzopardi K, Day S, Yeow S, Ranganathan S, et al. Australian respiratory early surveillance team for cystic fibrosis (AREST CF). A bacterial stimulation assay for bronchoalveolar lavage immune cells from young chil- dren with cystic fibrosis. Scand J Immunol 2021; 94(1): e13040. https://doi.org/10.1111/sji.13040*
8. *
  Steinke JW, Lawrence MG, Teague WG, Braciale TJ, Patrie JT, Borish L. Bronchoalveolar lavage cytokine patterns in children with severe neutrophilic and paucigranulocytic asthma. J Allergy Clin Immunol 2021; 147(2): 686-93.e3. https://doi.org/10.1016/j.jaci.2020.05.039*
9. *
  Choo R, Anantham D. Role of bronchoalveolar lavage in the management of immunocompromised patients with pulmonary infiltrates. Ann Transl Med 2019; 7(3): 49. https://doi.org/10.21037/atm.2019.01.21*
10. *
  Wong JCP, Hon KLE, Leung KKY, Qian S, Leung AKC. Diagnostic yield of bronchoalveolar lavage in immuno- compromised children. J Trop Pediatr 2021; 67(1): fmaa131. https://doi.org/10.1093/tropej/fmaa131*
11. *
  Jain K, Wainwright C, Smyth AR. Bronchoscopy-guided antimicrobial therapy for cystic fibrosis. Cochrane Database Syst Rev 2013; 23(12): CD009530. https://doi. org/10.1002/14651858.CD009530.pub2*
12. *
  Keil O, Schwerk N. Foreign body aspiration in children - is safe and flexible. Curr Opin Anaesthesiol 2023; 36(3): 334-9.https://doi.org/10.1097/ACO.0000000000001251*
13. *
  Gokdemir Y, Cakir E, Kut A, Erdem E, Karadag B, Ersu R, et al. Bronchoscopic evaluation of unexplained recurrent and persistent pneumonia in children. J Paediatr Child Health 2013; 49(3): E204-7. https://doi.org/10.1111/ jpc.12124*
14. *
  Ferraro VA, Baraldi E, Stabinger D, Zamunaro A, Zanconato S, Carraro S. Pediatric flexible bronchoscopy: A single-cen- ter report. Pediatr Pulmonol 2021; 56(8): 2634-41. https://doi.org/10.1002/ppul.25458*
15. *
  Morice AH, Millqvist E, Bieksiene K, Birring SS, Dicpinigaitis P, Ribas CD, et al. ERS guidelines on the diagnosis and treatment of chronic cough in adults and children. Eur Respir J 2020; 55(1): 1901136. https://doi. org/10.1183/13993003.01136-2019*
16. *
  De Schutter I, De Wachter E, Crokaert F, Verhaegen J, Soetens O, Piérard D, et al. Microbiology of bronchoalve- olar lavage fluid in children with acute nonresponding or recurrent community-acquired pneumonia: Identification of nontypeable Haemophilus influenzae as a major patho- gen. Clin Infect Dis 2011; 52(12): 1437-44. https://doi. org/10.1093/cid/cir235*
17. *
  De Baets F, De Schutter I, Aarts C, Haerynck F, Van Daele S, De Wachter E, et al. Inflammation and bronchoalveolar lavage culture in children with persistent respiratory symptoms. Eur Respir J 2012; 39(2): 392-5. https://doi. org/10.1183/09031936.00035111*
18. *
  Pietra A, Markiewicz M, Mielnik-Niedzielska G. Rigid bronchoscopy in foreign body aspiration diagnosis and treatment in children. Children (Basel) 2021; 8(12): 1206. https://doi.org/10.3390/children8121206*
19. *
  Efrati O, Gonik U, Bielorai B, Moses M, Neumann Y, Szeinberg A, et al. Fiberoptic bronchoscopy and broncho- alveolar lavage for the evaluation of pulmonary disease in children with primary immunodeficiency and cancer. Pediatr Blood Cancer 2007; 4. https://doi.org/10.1002/ pbc.20784*
20. *
  Nussbaum E. Pediatric fiberoptic bronchoscopy: Clinical experience with 2,836 bronchoscopies. Pediatr Crit Care Med 2002; 3(2): 171-6. https://doi. org/10.1097/00130478-200204000-00015*
21. *
  Schramm D, Yu Y, Wiemers A, Vossen C, Snijders D, Krivec U, et al. Pediatric flexible and rigid bronchoscopy in European centers-Availability and current practice. Pediatr Pulmonol 2017; 52(11): 1502-8. https://doi.org/10.1002/ ppul.23823*
22. *
  Terkawi RS, Altirkawi KA, Terkawi AS, Mukhtar G, Al-Shamrani A. Flexible bronchoscopy in children: Utility and complications. Int J Pediatr Adolesc Med 2016; 3(1): 18-27. https://doi.org/10.1016/j.ijpam.2015.12.003*
23. *
  Fonseca MT, Camargos PA, Abou Taam R, Le Bourgeois M, Scheinmann P, de Blic J. Incidence rate and factors related to post-bronchoalveolar lavage fever in children. Respiration 2007; 74(6): 6538. https://doi. org/10.1159/000107737*

